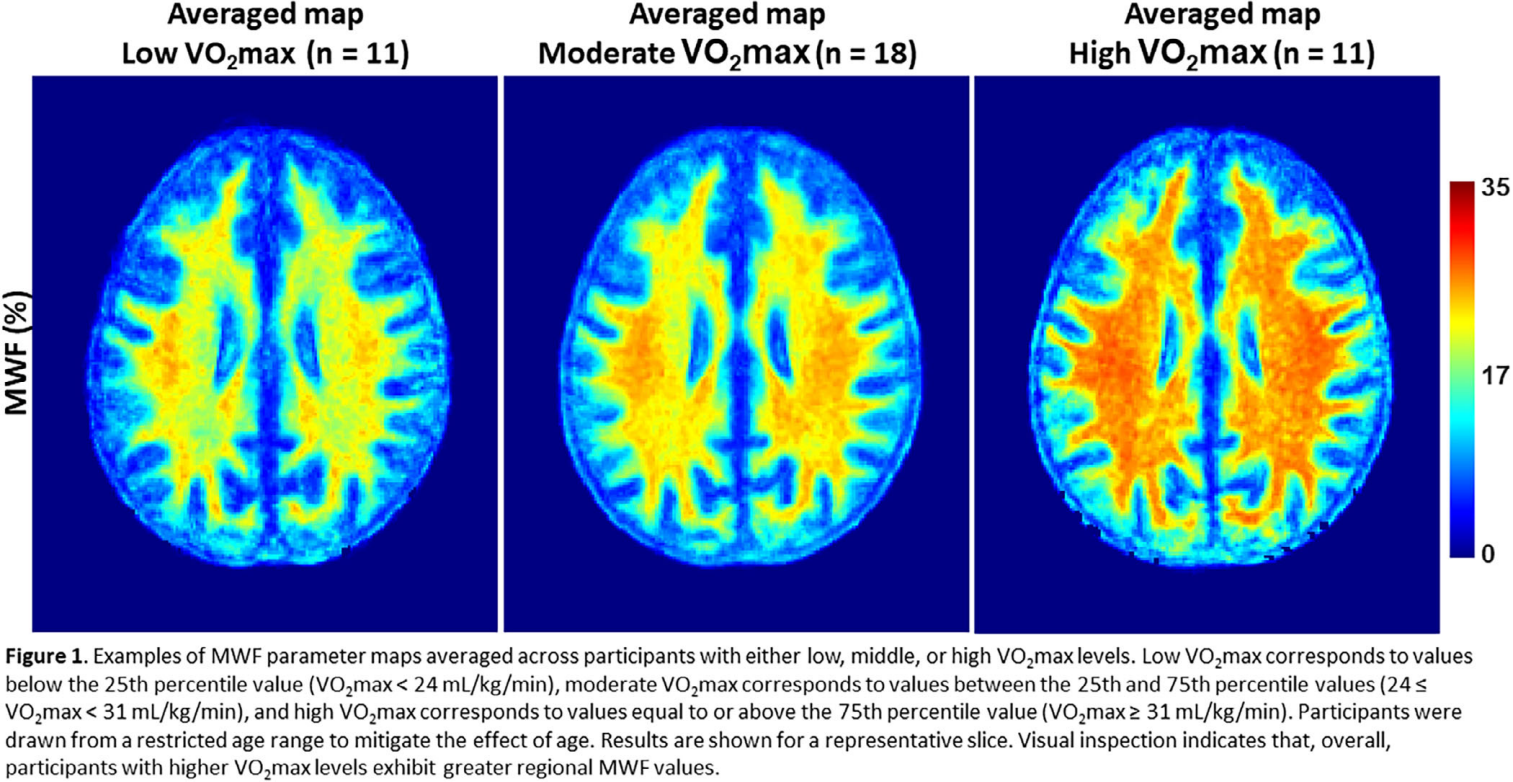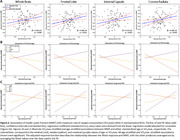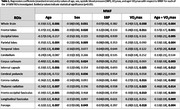# Higher cardiorespiratory fitness is associated with higher cerebral myelination in normative aging

**DOI:** 10.1002/alz.086203

**Published:** 2025-01-09

**Authors:** Mary E Faulkner, Zhaoyuan Gong, Murat Bilgel, John P Laporte, Alex Guo, Jonghyun Bae, Palchamy Elango, Christopher M Bergeron, Mustapha Bouhrara

**Affiliations:** ^1^ National Institute on Aging, Baltimore, MD USA; ^2^ National Institute on Aging, National Institutes of Health, Baltimore, MD USA; ^3^ Laboratory of Clinical Investigation, National Institute on Aging, Intramural Research Program, Baltimore, MD USA

## Abstract

**Background:**

Age‐related white matter (WM) deterioration has been associated with several neurodegenerative diseases, including Alzheimer’s disease. Studies suggest that cardiorespiratory fitness (CRF) is important in preserving WM structure and function. However, clinical investigations of the relationship between myelin integrity and CRF are lacking. In this study, we evaluated the association between CRF, assessed by the maximum rate of oxygen consumption (VO_2_max), and cerebral myelin content, defined by myelin water fraction (MWF), throughout the adult lifespan. Understanding the mechanisms of WM degeneration is crucial for developing targeted interventions to maintain cognitive health into late life.

**Method:**

After excluding subjects with cognitive impairments or unexploitable MRI data, a total of 110 cognitively unimpaired participants, ranging in age from 22 to 94 years, were drawn from the BLSA and GESTALT studies. Each participant underwent our BMC‐mcDESPOT MRI protocol for whole brain MWF mapping. VO_2_max was assessed using graded maximal treadmill test as per our established protocol. Fourteen WM ROIs were defined from the MNI structural atlas. For each ROI, a linear regression model adjusted for relevant covariates (Table 1) was used to determine whether 1) VO_2_max is significantly correlated with MWF, and 2) the strength of the correlation between VO_2_max and MWF depends on age (age×VO_2_max interaction). Continuous variables were z‐scored.

**Result:**

Higher VO_2_max was significantly associated with higher MWF (Figure 1). This association was strongest in brain regions susceptible to early degeneration (Table 1), including the frontal lobes (β=0.28, p=0.011) and white matter tracts, such as the internal capsule (β=0.27, p=0.025) and corona radiata (β=0.27, p=0.016). Further, the interaction between age and VO_2_max exhibited i) a steeper positive slope in the older age group, suggesting that the effect of VO_2_max on MWF is stronger at older ages, and ii) a negative slope in the lower VO_2_max group, indicating that lower VO_2_max levels are associated with more rapid demyelination with advancing age (Figure 2 & Table 1).

**Conclusion:**

These original findings provide the first evidence that high CRF is important for the preservation of myelin integrity and slowing of age‐related neurodegeneration, laying the groundwork for further investigations including longitudinal and interventional studies.